# Case report: The physiology of a preventable tragedy –Near death in a hot tub

**DOI:** 10.1002/ccr3.4951

**Published:** 2021-10-26

**Authors:** Bruce E. Becker

**Affiliations:** ^1^ University of Washington School of Medicine Bend Oregon USA

**Keywords:** anoxia, aquatic immersion, hot tubs, hyperthermia, pediatric, traumatic brain injury

## Abstract

Hyperthermia in children is a known risk within enclosed vehicles. Exposure to an overheated hot tub poses a real risk in children due to unique pediatric physiology. Medical and aquatic professionals should understand the risk and mitigation strategies.

## Graphical abstract

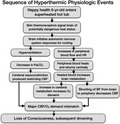

## 1 THE CASE STUDY

Hot tubs are a common source of relaxation and recreation. While recommendations exist for tub temperatures and guidelines regarding their use by children and adults with potential risk factors, these are commonly ignored. Environmental factors such as solar gain may dramatically alter tub temperatures well beyond those set by the regulating thermostat, as commercial hot tubs have no cooling mechanisms and tub temperatures may rise well beyond safe ranges. This article describes a situation involving an overheated hot tub with tragic consequences, referencing the underlying physiology, and describing means of prevention of such adverse consequences.

Mid‐afternoon on a very hot day in August in the southern United States, a mother, her two children, and a friend entered a hotel water park, anticipating a relaxing afternoon in the water playground. This was not intended as an area for adults except as observers and guardians for their children. It was filled with several slides, and many spray venues, both overhead and ground level. In a corner of the area was a circular hot tub, a carry‐over from the previous facility which was more adult‐centric with a full pool.

**FIGURE 1:**
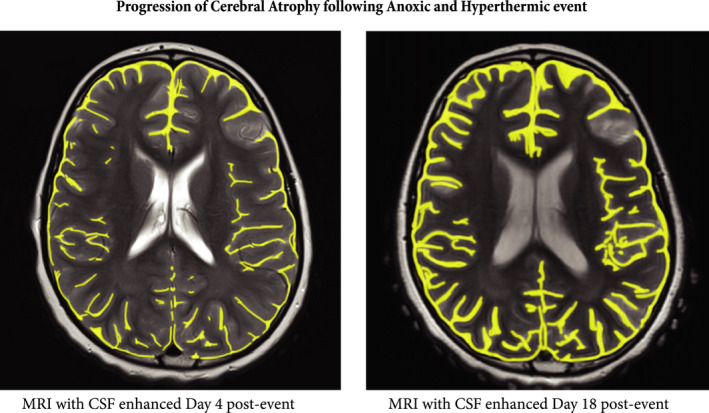
Cerebral atrophy following anoxia and hyperthermia here

The temperature was 100° on this cloudless day following a lengthy period of extended heat wave. The environment looked very appealing. The mother and her children sat on recliners in the vicinity of the hot tub which was empty at the time and still. The mother assumed because the jets were not on that the tub had been turned off. The younger children asked whether they could go into the hot tub. The mother consented, and the two youngest entered the tub using the stairs. The youngest child excited to play in the spray features, and the 6‐year‐old, a skilled swimmer remained in the tub seated on the bench edge inside the tub with water nearly reaching her neck. The mother was observing all of the children across the area while her oldest child, a teenaged brother, departed to change into his swimsuit. As he returned from the changing room, he saw that his sister was face down in the tub and called to his mother. The mother and brother immediately pulled the unconscious girl from the tub onto the hot concrete pool deck, calling for help. A nurse visiting the pool area was alerted to the situation, ran to the child, and assessed her, finding no pulse or respiration. She asked for a towel to be placed under her and immediately initiated CPR with another medical professional pool guest assisting. 911 was called. After several cycles of CPR, the young girl began breathing and her pulse returned. She vomited frothy material, but she remained unconscious. EMTs arrived and assessed the situation, supplying oxygen via facemask and noting labored respiration with lungs sounding wet. They recorded that her skin was very hot to touch and that the young girl was unconscious with a Glasgow score of 8. She was then evacuated on a gurney to the closest emergency room after their arrival. In route, she had a seizure, without any prior seizure history. Her temperature on ED arrival was 104.7°F. First and second degree burns were noted on her back, upper arms, and calves were noted from lying on extremely hot concrete during her resuscitation. She remained very febrile in hospital for a period of days with very low consciousness scores. Medical imaging demonstrated significant brain injury. After a series of transfers between hospitals over a period of several weeks, she was discharged home with very apparent severe brain damage. Years later, she remains cognitively very impaired (Figure 1).

## 2 THE PHYSICAL ENVIRONMENT

The hot tub thermostat had been set at 102°F, although on the day prior and subsequently during the early morning, the County inspector had measured the actual tub temperature at 104°F. This event happened in mid‐late afternoon, allowing solar gain to heat the hot tub well beyond 104°F. A systems engineer using finite element analysis estimated the tub temperature to be between 106° and 109°F on the day and time of the event. Using the same finite element analysis methodology, the temperature of the surrounding concrete was estimated at 139°F. As a consequence of the solar gain on both tub temperature and concrete, this was a particularly dangerous environment for the risk of hyperthermia.

## 3 THE PHYSIOLOGY

### 3.1 Introduction

Children are physiologically quite different from adults. Because of their smaller body mass, environmental temperatures produce more significant changes, and that smaller mass raises core temperatures more rapidly. Children have a smaller total blood volume in relation to body mass than adults. Children have more rapid autonomic nervous system responses, sweating more rapidly, vasodilating more quickly, and altering systemic blood pressure due to greater vascular compliance. As a consequence, they are both more fragile and have a wider range of physiologic responses to cope with environmental circumstances.

### 3.2 Hyperthermia

Hyperthermia in children is quite common, potentially occurring post‐exercise, and environmentally, such as when inside enclosed cars.^1^ The number of hot tub thermal injuries is unknown. That said, at a temperature of 34°C, the only effective means of body cooling is through the evaporation of perspiration, and within a hot water environment, there is no evaporative loss.^2^, ^3^ Since there are no effective biologic means of temperature reduction, hot water immersion poses a substantial potential physiologic risk for both children and adults. Children are more susceptible to hyperthermia for several important reasons. Children's skin is thinner than adults, and their surface area to body mass is much higher.^4^ During heating, children also have elevated skin temperatures when compared to adults, indicating higher levels of peripheral vasodilatation.^4^ This allows faster heat transport (but also faster heat return as in this case). Children also have a lower total blood volume per body mass than adults, presenting decreased cooling opportunity and also a potential for decreased central and brain blood flow when blood is diverted from central circulation to the periphery.^4^ The combination of these two vulnerabilities means that children raise their core temperatures much faster than adults and thus face a faster and higher risk of hyperthermia, especially when the peripheral circulatory increase only serves to return heated blood into the circulation.^5^

### 3.3 Cardiac output

Simple aquatic immersion produces a long‐recognized increase in cardiac output.^6^, ^7^, ^8^, ^9^, ^10^ This has not been studied in children during immersion. In land‐based exercise in warm environments, children do show increase in cardiac output but to a lesser degree than in adults.^4^ In a warm or hot environment, the first response of the body is to increase peripheral circulation, especially to skin surfaces to facilitate sweating.^3^ The magnitude of this response is substantial, increasing peripheral circulation 15 cm^3^/min for each 0.01°C rise in body temperature, with potentially a 25‐fold increase in cutaneous blood flow.^3^, ^11^ Children have higher peripheral blood flow than adults in comparable high temperatures.^4^ While core temperature rises far more slowly than skin temperature, it is highly likely that the initial neurologic signaling from the skin is what triggers this response because this happens far quicker to protect thermal homeostasis. Work done in our laboratory demonstrated nearly a 1200% increase in peripheral circulation within the first 4 minutes of warm water (39°C) immersion in young subjects, nearly double that seen in an older cohort.^12^, ^13^ This increase in peripheral blood flow is facilitated by the increase in cardiac output, but the redistribution of blood reduces the blood flow to central structures and the brain.^3^ The speed with which this occurred was dramatic, with immediate increase in blood flow within seconds. In a warm water environment, the heated blood returns uncooled and even heated to the brain, signaling a demand for yet more peripheral blood flow, creating a vicious cycle until maximal peripheral flow is achieved.^3^

### 3.4 Brain metabolism and blood flow

Despite the brain occupying only 2%–3% of total body mass, the brain consumes around 20% of total body oxygen consumption.^11^ The brain has a very high overall metabolic rate, a single neuron consuming many hundred‐fold the power consumption of an average body cell.^14^ This high level of metabolism causes the brain to normally operate at temperatures 0.3–0.8°C above core temperature, and this figure can rise substantially during increased demand periods, and particularly during hyperthermia.^14^ This temperature increase is normally managed by cerebral blood flow (CBF) bringing in cooler blood, so that there is a differential between arterial inflow and warmed venous outflow. In addition to providing oxygen and other needs to the brain, it also transports heat away from it.^15^ Recent publications on young adults have documented an increase in the cerebral metabolic rate of oxygen consumption (CMRO_2_) of 10%–20% during hyperthermia.^15^, ^16^ Studies have not been done in children on this issue due to ethical constraints on pediatric research.

Simple immersion and immersed exercise increase brain blood flow by significant amounts, although human studies to date have not yet included children as noted above.^17^, ^18^, ^19^ The studies demonstrating this used thermoneutral water temperatures rather than warm water. The normal increase in CBF from thermoneutral immersion is overwhelmed in the face of hyperthermia. The normal physiologic response to hyperthermia uses two mechanisms of cooling: hyperventilation and increasing cerebral blood flow. Hyperventilation typically produces a reduction in arterial CO_2_ pressure (PaCO_2_), which causes cerebral vasoconstriction.^11^ This cerebral vasoconstriction is one factor in reducing CBF. A 2020 study of passive hyperthermia corrected for this respiratory alkalosis by using an end‐tidal forcing apparatus to correct PaCO2 to normothermic levels and found essentially the same effects on cerebral metabolism elevation and cerebral blood flow reduction.^16^ Furthermore, the diversion of blood from the brain to peripheral circulation in an attempt to promote skin cooling produces a reduction in CBF, an effect magnified in children due to their smaller total blood volume. As a consequence of a reduction in cerebral blood flow with a simultaneous increase in cerebral metabolism, there is very real potential for cellular injury to brain cells. When brain temperatures reach 40° to 41°C, particular cell lines are damaged and when reaching 42°C, irreversible damage occurs.^3^, ^20^ The duration of hyperthermia is particularly relevant in promoting cellular survival, so aggressive rapid cooling measures are essential.^21^

### 3.5 Syncope

Syncope can result during such a loss of CBF and is exacerbated by the increased O_2_ demands of a hypermetabolic brain.^11^ Disorders of cerebral function and syncope can occur with decreases of CBF below 60% of normal. When CBF drops to only 40% of normal, unconsciousness occurs in seconds.^3^

### 3.6 Drowning

Normally respiration does not cease with syncope. On land when the person sustains a collapse, cerebral blood flow increases, resulting in return to consciousness. When syncope occurs in an aquatic environment, the body attempts to breathe, but instead of taking in air, water enters the airway, triggering laryngospasm and consequent inability to breathe. This initiates the drowning process.^22^ With increasing hypoxia, the laryngospasm abates and further aspiration of water may occur. This often results in post‐drowning pneumonia in 30%–50% of drowning survivors.^22^ Such was the case in the case report above. When drowning occurs in cold water environments, the body and brain may cool sufficiently to prevent massive cerebral damage, but in heated water, the increase in brain metabolism combined with hypoxia may be catastrophic.^22^

### 3.7 Hyperthermia following cerebral injury

In the case report following immersion in an excessively heated body of water, the initial hyperthermia was a passive consequence of that environment. It was followed by a prolonged period of resuscitation while laying on 139°C concrete for at least 20 min, resulting in burns to her back and extremities. As soon as her heart resumed function following CPR, the superheated blood in her trunk and extremities was returned to her central circulation and brain. Once she was hospitalized, aggressive attempts to cool were made. Over the following week or more, her temperature fluctuated dramatically. Traumatic brain injury alone is associated with aseptic hyperthermia in 30%–70% of pediatric cases and is closely associated with the magnitude of brain injury and subsequent recovery.^20^, ^23^ As a result, current best practice is to provide cooling to normothermic or lower levels during the period following resuscitation of cardiac arrest or hospital care following TBI.^23^ The longer the brain is allowed to remain in a hypermetabolic state, the poorer the ultimate recovery.^23^

### 3.8 Brain Injury

The primary temperature‐sensitive elements of neural cells are mitochondrial and plasma membranes.^20^ The protein structures within these structures become disrupted and may undergo irreversible abnormalities at temperatures even above 40°C.^20^ This is potentiated by hypoxia.^20^ Children with their rapidly developing brain cells may be at greater risk than adults, and while some cell death occurs quickly, other cells may undergo a prolonged period of apoptosis.^20^ Purkinje cells seem to be the most at risk, showing cellular changes to the greatest extent.^20^ As a consequence, the cerebellum may undergo the greatest damage with loss of motor control.^20^ This has been the case in the subject described above, with on‐going deficits in gait function, bimanual motor control, and coordination.

## 4 SUMMARY OF THE PHYSIOLOGY

**FIGURE 2:**
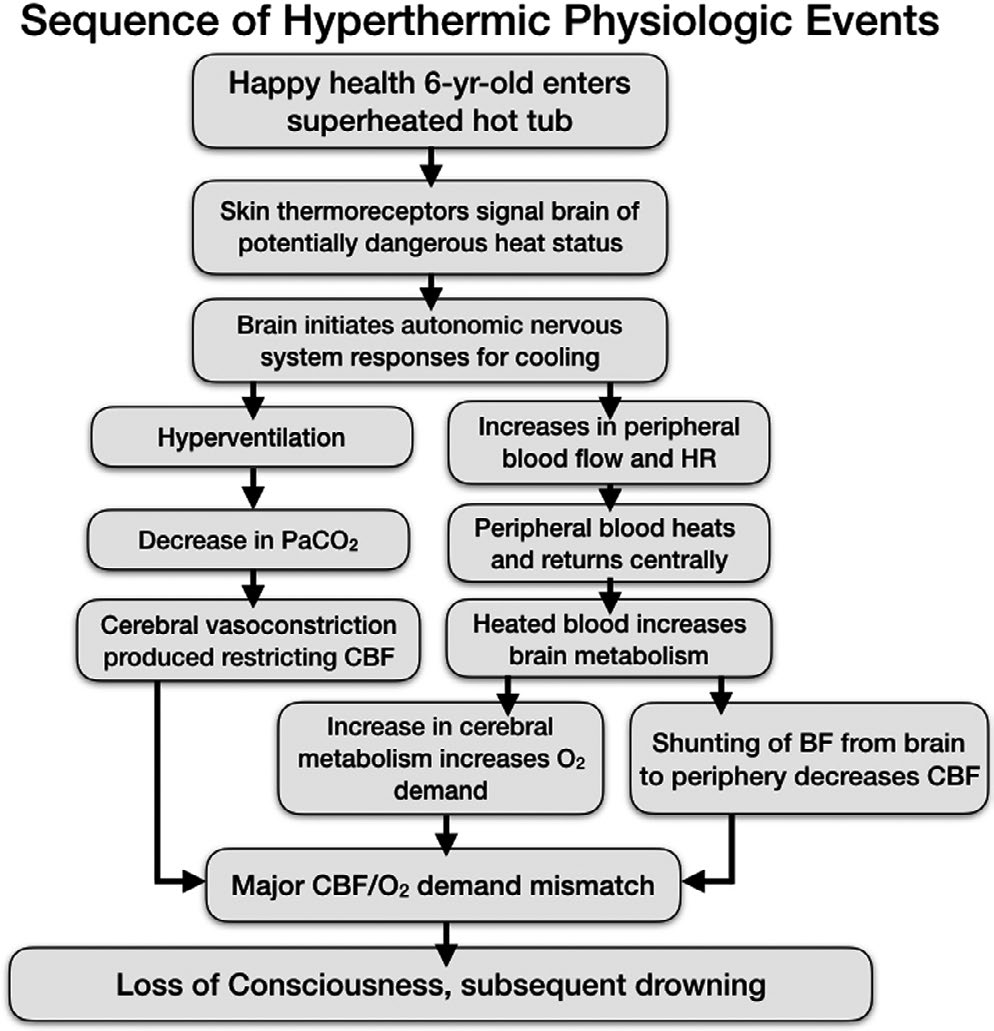
Schematic of physiologic events here

The child enters into an overheated hot tub, conscious, and alert. Her skin temperature sensors signal the brain to immediately initiate systemic cooling through peripheral vasodilatation and sweating. The overheated water prevents cooling and indeed adds to it. The heated blood returns into central circulation, increasing brain temperature. More central blood is sent to the periphery in an attempt to increase cooling. Brain metabolism and oxygen demand increase with temperature. Central blood flow to the brain decreases due to the shunting to the periphery despite the increase in brain metabolism. Syncope results from the CMRO_2_ to O_2_ availability mismatch, resulting in the child slumping forward into the water face underwater. Drowning begins, producing anoxia along with increasing blood temperature. The child is recovered from the water and laid on the very hot concrete during resuscitation. Peripheral blood heats further, and when cardiac function is restored, superheated blood returns from the periphery to the brain, further increasing cerebral metabolism and anoxic cerebral injury (Figure 2).

## 5 DISCUSSION

The frequency of the events described above is unknown; there is no national registry of pediatric drownings which includes causation.^24^ Drowning is the leading cause of injury and death in the USA between the ages of 1–4 and the 3rd leading cause in the ages from 5–19 years of age.^24^ Supervision remains the most essential means of protection. The Model Aquatic Health Code developed by the CDC is both based on science and standards for safety of operation. The code states maximum temperature “shall not exceed 104°F.”^25^ The policy statement from the American Academy of Pediatrics referenced fails to mention hyperthermia risk from hot tub use.^24^ In the case described, these standards were not met: the hot tub temperature was substantially in excess of this code and in actuality signage within the pool area failed to describe risks of overheating. There were no depth markers thus leaving visitors little forewarning of such risk. While the age of the child involved was over the age of 5, below which hot tub use is warned against by the CDC, she was close enough to have raised concerns. However, using age 5 as an arbitrary guideline ignores the many situations that are age‐unrelated, such as growth disturbances, physical or cognitive disabilities, and underlying diseases which may have added potential risks. The presence of a hot tub with heater set to 104°F did not include any means of cooling should that temperature be exceeded, and it is unclear as to whether there were any means of alerting operators if this temperature was exceeded. There were no thermometers in the hot tub. While the mother did not accompany the child into the tub, she had believed that the tub was off, as the jets were not working, and her child was a skilled swimmer.

Hyperthermic syncope can occur suddenly in both children and adults, with potentially catastrophic results. It is poorly recognized as a risk by medical professionals, aquatic facility supervisors, lifeguards, and the general public. This case demonstrated the risks, the unfortunate outcome, and the underlying physiology behind those risks.

## 6 PREVENTION

Far broader awareness of the risks of hyperthermia is needed by medical professionals, the lay public, and aquatic facility owners. This event could have been prevented by a mother alerted to the risks presented by an overheated hot tub, made aware by signage, and by available thermometers showing actual tub temperature. The aquatic attendant should have been aware of both the tub temperature and the risks thus presented, and equipped to manage adverse events. Facility management should have made adaptations to the hot tub to avoid such risks through appropriately covering the area to reduce solar gain, and setting the temperature no higher than 100°F, as described in the Model Aquatic Health Code. An Emergency Action Plan specific to this set of circumstances should have been available and part of employee training. Facility management should also have had better oversight of the tub vicinity than a single attendant over the entire large area.

## 7 EMERGENCY ACTION PLAN ADDITIONS FOR PUBLIC FACILITIES

1. Highly legible signage alerting public to the risks of hyperthermia should be prominently posted adjacent to hot tubs.
2. All staff overseeing such sites should have both training in awareness of and management for hyperthermic events.
3. All hot tubs should have temperature oversight with thermometers readily visible and accessible within the tubs.
4. Insulating pads and cooling mechanisms (ice) should be readily available in case of such events.

## CONFLICT OF INTEREST

I have no real or potential conflicts of interest.

## Author Contributions

The author confirms sole responsibility for the manuscript preparation, referencing, and submission processes. As the physician charged with understanding the physiology behind this tragic event, the existing medical literature was insufficiently comprehensive, prompting this paper.

## CONSENT

No identifying information was included in the manuscript. As the patient is a minor, her guardian mother has given both verbal and written permission for the article publication.

## Data Availability

Data sharing not applicable—no new data generated.
